# A century of variation in the dependence of Greenland iceberg calving on ice sheet surface mass balance and regional climate change

**DOI:** 10.1098/rspa.2013.0662

**Published:** 2014-06-08

**Authors:** G. R. Bigg, H. L. Wei, D. J. Wilton, Y. Zhao, S. A. Billings, E. Hanna, V. Kadirkamanathan

**Affiliations:** 1 Department of Geography, University of Sheffield, Sheffield S10 2TN, UK; 2 Department of Automatic Control and Systems Engineering, University of Sheffield, Sheffield S10 2TN, UK

**Keywords:** nonlinear auto-regressive moving average with exogenous modelling, Greenland icebergs, coupled ocean–iceberg model, twentieth century climate variation

## Abstract

Iceberg calving is a major component of the total mass balance of the Greenland ice sheet (GrIS). A century-long record of Greenland icebergs comes from the International Ice Patrol's record of icebergs (I48N) passing latitude 48° N, off Newfoundland. I48N exhibits strong interannual variability, with a significant increase in amplitude over recent decades. In this study, we show, through a combination of nonlinear system identification and coupled ocean–iceberg modelling, that I48N's variability is predominantly caused by fluctuation in GrIS calving discharge rather than open ocean iceberg melting. We also demonstrate that the episodic variation in iceberg discharge is strongly linked to a nonlinear combination of recent changes in the surface mass balance (SMB) of the GrIS and regional atmospheric and oceanic climate variability, on the scale of the previous 1–3 years, with the dominant causal mechanism shifting between glaciological (SMB) and climatic (ocean temperature) over time. We suggest that this is a change in whether glacial run-off or under-ice melting is dominant, respectively. We also suggest that GrIS calving discharge is episodic on at least a regional scale and has recently been increasing significantly, largely as a result of west Greenland sources.

## 1. Introduction

**Figure 1.**
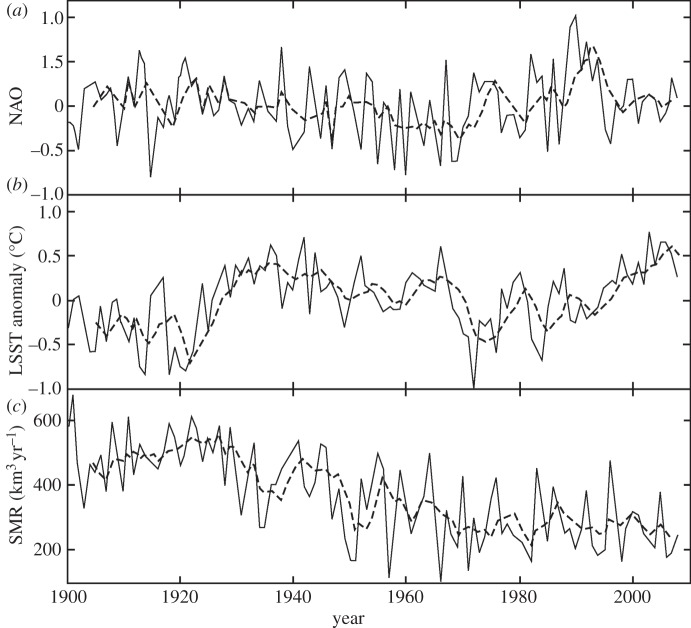
Annual GrIS SMB (*c*), in km^3^ yr^−1^, LSST (*b*) anomaly, in °C, and North Atlantic Oscillation (NAO; *a*), standardized to 1 s.d., for 1900–2008. The solid line in each panel shows the annual average, and the dashed line is a 5 year running mean. See §2*a* for a description of the origin of these three datasets.

The iceberg calving flux is a major component of the total mass balance of the Greenland ice sheet (GrIS). Past work, using a variety of approaches, has found that one of the components of the GrIS total mass balance, namely the surface mass balance (SMB; figure 1) of the GrIS, has high interannual variability, but with two periods of significant decline over the past century, during 1930–1960 [1] and again in the last decade [1,2]. By contrast, current estimates of one measure of the other key component, the solid ice loss, since the early 1990s, namely the ice flux across the GrIS grounding line, suggest this latter function varies much more smoothly on the ice sheet scale [2] but has increased by approximately 20% since the 1990s. These ice loss estimates exhibit no linear correlation with SMB, except when smoothed and considered over the long term [3]. Three questions are raised by these analyses. Is the iceberg discharge, rather than grounding line ice flux, smoothly varying, given the large background flux, the doubling in estimates of its size in the last decade (cf. [2] with [4–6]) and historical [7] and recent palaeoceanographic [8] records of strong variability? How has this discharge varied over longer time scales than can be measured from satellites? What are the major environmental variables driving these changes?

**Figure 2.**
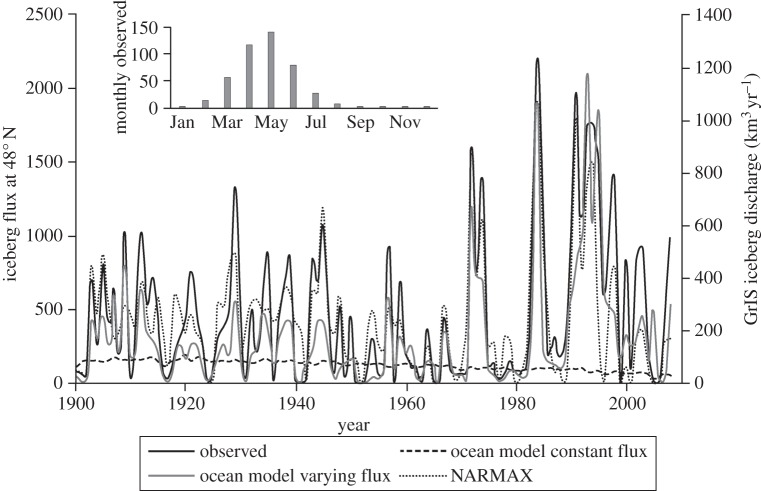
Iceberg numbers at 48° N. Yearly totals of the observed iceberg number (in black; I48N), the ocean model's iceberg flux (unvarying discharge and discharge scaled as equation (2.1)) and the NARMAX model's fit to I48N. Right axis shows GrIS calving discharge with I48N used as proxy (in Gt yr^−1^ or km^3^ yr^−1^). The inset shows I48N's mean annual cycle.

Monthly, counts of iceberg numbers observed south of 48° N in the western North Atlantic have been compiled by the United States Coast Guard since 1913, absorbing previous reports to the US Hydrographic Service back to 1900. While these numbers have always been acknowledged as estimates [9] and come from a range of platforms evolving over time (ships to aircraft, radar, the use of models and more recently satellites [10]), the series (I48N) is the only long-term estimate of iceberg numbers in the northwest Atlantic available. I48N has a strong, and regular, seasonal cycle (figure 2), with a pronounced peak in numbers in spring to early summer. This is likely to be due to a combination of seasonal peaks in discharge [11], a delay effect from the release of icebergs being trapped in winter sea ice [12] and varying travel paths [13]. However, interannual variability in I48N will be due to a combination of variations in the calving flux, overwhelmingly from Greenland [12], as well as climate fluctuations over the northwest Atlantic. It is known that the former can be very variable from year to year for individual glaciers [8,14,15]. However, the Labrador Sea climate can also change very quickly [16]. Therefore, in this study we explore the long-term variability in iceberg discharge from the GrIS through considering I48N using a combination of ocean–iceberg and nonlinear auto-regressive moving average with exogenous input (NARMAX) system identification modelling both to separate the discharge signal in I48N from the mean ocean–atmospheric interactions following calving and to understand the processes and time scales involved in its variation. The NARMAX approach is described below in the Material and methods, but, briefly, it is a modelling framework which allows the user to construct linear or nonlinear dynamic models between inputs (exogenous variables) and outputs (auto-regressive variables) in the presence of coloured and nonlinear noise.

## 2. Material and methods

### (a) Data

I48N came from the International Ice Patrol's (IIP) iceberg counts over 1900–2008 (http://www.navcen.uscg.gov/pdf/iip/International_Ice_Patrols_Iceberg_Counts_1900_to_2011.pdf). They are monthly counts of all separate icebergs larger than a visible length of 5 m observed south of a line extending along 48° N from the Newfoundland coast to approximately 40° W (see http://www.ec.gc.ca/glaces-ice/ for eastward extent). By the nature of the observations, they will include icebergs from any origin, but are not able to distinguish the calving origin of individual icebergs. A full discussion of this time series is contained in [9,10,12,17], with discussion of its implications for the Greenland iceberg flux found in [12,18]. It has always been the purpose of the IIP to provide ice hazard information to the major shipping routes into eastern North America [9]. Missing significant icebergs entering these shipping lanes has potentially serious consequences, but since the institution of the IIP there have been no serious incidents with icebergs, for vessels heeding IIP warnings [9]. This fact alone speaks to the thoroughness of the surveys leading to the I48N compilation.

The Greenland SMB was based on a positive degree day run-off/retention model [1], using a 5 km grid [19]. The monthly North Atlantic Oscillation (NAO; [20]) time series is principally component-based. The Labrador Sea surface temperature (LSST) comes from averaging the Kaplan v2 SST [21], over the Labrador Sea area. The daily forcing of both the ocean–iceberg–sea-ice model and the SMB model comes from the relevant fields of the twentieth century reanalysis (20CR; [22]). Note, however, that the SMB series is a composite based on the 20CR (1871–1957) and European Centre for Medium-Range Weather Forecasts (ECMWF; ERA40 reanalysis 1958–2001 plus ECMWF operational analysis 2002–2010) temperature, and precipitation datasets that were calibrated/validated against *in situ* data and spliced together [1]. The composite SMB series agrees very well with independent SMB series for the common overlap period of 1958–2010 (see fig. 9 of [1]).

### (b) Ocean–iceberg model

The ocean component of the coupled ocean–iceberg–sea-ice model [23] is a curvilinear general circulation ocean model, with its North Pole displaced to central Greenland to increase horizontal resolution in the North Atlantic and Arctic [24], in this case to less than 0.5° locally around Greenland, and whose sea-ice component is a thermodynamic model with simple advection. To force the ocean–iceberg model on a daily time step over the period covering the same time span as that of the observed iceberg flux record at 48° N, we use data from the 20CR. This dataset provides estimates of global atmospheric variables at a 3 hourly temporal, and 2° spatial, resolution. It is derived from surface pressure observations, with observed sea surface temperatures and sea-ice distributions as reanalysis boundary conditions. We calculated daily averages of the variables required for the ocean–iceberg model's heat, fresh water and wind fluxes at each model gridpoint. A discussion of the ability of the model to reasonably reproduce mean circulation fields is given in [18,23,24]; the strong variability stemming from daily forcing is illustrated in [25].

The coupled iceberg component is a dynamical and thermo-dynamical iceberg trajectory model [26,27], with a range of iceberg sizes released from the main Northern Hemisphere calving sites [23], carrying a weighting scaled by an estimated iceberg flux. This underestimates the total flux from Greenland by somewhat more than half, because it uses only the main 20 release sites and does not include icebergs from the many small tidewater glaciers, although a range of sources from around the entire Greenland coast are used (see fig. 2 of [23] for both the location and relative sizes of discharges). In addition, estimates of the total GrIS calving or grounding line discharge have changed radically in recent years, from around 200 Gt yr^−1^ [4,28,29] to over 500 Gt yr^−1^ [2]. Some of this change is the result of improved methods of estimation, and some is the result of likely recent increases in mean flux.

The iceberg model has been extensively tested previously in the Arctic [26], Antarctic [30] and glacial climatic conditions [31–33]. Its behaviour due to variation of the various forcings to which icebergs are subject has been fully explored [27]. The iceberg model has shown itself to be robust in capturing the mean trajectories, limits and densities of icebergs.

The first experiment uses the same flux of icebergs each year, but with a regional variation in release proportions through the year. South of 64° N, 15% of the annual flux is released in October, 75% in January and 10% in April, to match when sea ice breaks up from southern Greenland fjords (from analysis of Landsat imagery), while north of this latitude the releases are split 15% in April, 75% in July and 10% in October to match peak calving times [11,15]. The second experiment used an annual total flux varying from year to year and scaled according to

2.1 $$D_{g}(t)=c\left(\frac{\text{I}48\text{N}(t^{\prime })}{\text{I}48{\text{N}}_{m}}\right)D_{\mathrm{gclim}},$$

where *D*_*g*_(*t*) is the annual flux for a given tidewater glacier, *t* is time in years, I48N_*m*_ is the mean observed I48N, *D*_gclim_ is the climatological mean annual discharge of the glacier [26], *t*^′^=*t* for glaciers south of 64° N with *t*^′^=*t*+1 for glaciers north of 64° N and *c* is a scaling parameter (270/96) to reflect the underestimate of the total GrIS calving discharge by the model sampling of release sites. There are few well-documented datasets of iceberg trajectories [13,34,35] but the model is consistent with these (e.g. electronic supplementary material, figures S1 and S2).

### (c) Nonlinear auto-regressive moving average with exogenous modelling

The NARMAX system identification model [36–38] uses a forward regression orthogonal least-squares algorithm to build models term by term from recorded datasets. This is achieved by using the error reduction ratio (ERR), which shows the contribution that each selected model term makes to the variance of the dependent variable (I48N here) expressed as a percentage, taking account of the noise in the data [38]. The NARMAX method searches through an initial library of model terms, which typically includes linear and nonlinear lagged variables, and selects the most significant terms to include in the final model. NARMAX methods have been widely applied to many problems across a wide range of scientific disciplines, such as engineering, modelling space weather, electroencephalography, visual systems in insects and many others (e.g. [39–41]; see ch. 14 of [38] for further examples).

Annual data of all independent and dependent variables were used to construct the NARMAX models. Three physical variables that will have an impact on melting processes near a glacier's marine termination are the SMB, representing the glacier's surface run-off/accumulation balance, a measure of ocean temperature (LSST), which affects under-ice melting, and an atmospheric circulation measure (NAO). In all cases, we have chosen large-scale quantities as we are considering mean calving behaviour over extensive areas of Greenland, rather than individual fjords. These three variables can be seen as measures of the three glacier retreat mechanisms recently proposed by Straneo *et al*. [42]. It is worth noting that previous work has shown that the Newfoundland sea-ice extent is strongly correlated with I48N [12]. In a separate study [43], we show that this correlation is not due to an independent process, but is linked to one or more of the three variables we use here for the NARMAX model.

Initial studies showed that auto-regressive terms did not contribute significantly to the models (that is, terms involving I48N itself); such terms were therefore excluded from the library of terms for the model fits. A wide range of potential lag terms extending over several years were also initially tested, but as the significant contributions consistently came from those under 4 years, this period was used to limit the possible number of terms for computational reasons. Similarly, the order of the polynomials principally contributing to the NARMAX models was explored through seeing which terms arose naturally from the NARMAX procedure. The most significant terms, considering their ERR values, were up to third order, so the final NARMAX model only allowed up to third-order polynomials.

In this work, a procedure with a 30 year sliding window, to allow for a temporally evolving solution, has been developed. A previous analysis using this sort of approach in electroencephalography is described in [41]. We did many initial studies on our dataset which are not discussed in this paper to save space (these are currently being prepared for publication). However, mathematical analysis showed that the information in the data is non-stationary and therefore the full dataset cannot be treated as a homogeneous system. We tried different length data windows to optimize the modelling of the time variation within the data and concluded that a 30 year window was the most appropriate. The window is moved through the data, so that each window's model is created from the data of that particular 30 year window. Time variation will therefore be tracked using this approach. This includes effects that are longer than the window length as these longer term changes will moderate the data through this and neighbouring windows in a way that the sliding window approach can track. Fitted values up to 1929 use the model variables of the first sliding window (1900–1929), but years thereafter use the evolving model from each successive sliding window finishing in the year fitted. An extensive set of sensitivity tests involving combinations of variables, and monthly versus annual data, were carried out. Here, we show only the full annual model, but its results are consistent with all the sensitivity tests. Note that a lag here of 0 years between variables does not eliminate the opportunity of cause followed by effect a few months later.

## 3. Results

### (a) Non-stochastic nature of I48N

It is first shown that I48N cannot be generated by a purely stochastic process, but should be treated as being forced by external inputs. To support this argument, we generated 100 surrogate datasets, denoted by I48N^(*s*)^, from the original raw monthly I48N data by using an amplitude adjusted Fourier-transformed surrogates algorithm [44,45]. For each of the surrogate datasets, a nonlinear auto-regressive moving average (NARMA) model of the form

**Figure 3.**
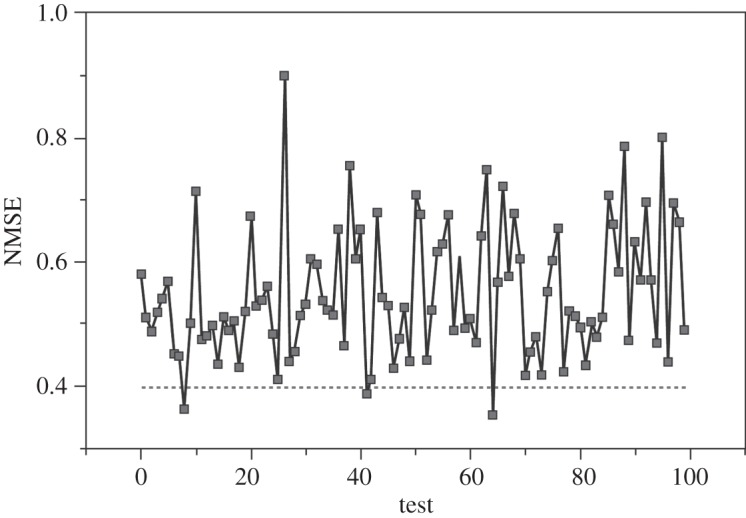
A comparison of NMSE values for the 100 surrogate tests (solid line) and the raw I48N data (dashed line).

3.1 $$\text{I}48{\text{N}}^{\left(s\right)}(t)=f^{\left(s\right)}(\text{I}48{\text{N}}^{\left(s\right)}(t-1),{\text{I48N}}^{\left(s\right)}(t-2),\ldots ,{\text{I48N}}^{\left(s\right)}(t-24),e(t-1),\ldots ,e(t-24))+e(t)$$

was identified, where *f*^(*s*)^(.) are nonlinear functions corresponding to the s-th surrogate, and *e*(*t*) is a noise sequence. The maximum nonlinear degree of the model terms was chosen to be 3. In order to show that I48N is not a purely stochastic process, we calculated the normalized mean squared error (NMSE) for each of the 100 surrogate models. Only three NMSE values out of the 100 surrogate tests are lower than that for the original data (figure 3). This result statistically shows that I48N cannot be a purely stochastic process, even taking into account possible stochastic errors in the variable, and seeking physical causes for its variation is a valid pursuit. For the rest of the paper, we use the annual averaged I48N for clarity.

### (b) Ocean modelling

Whether the variation of I48N is dominated by oceanic or glaciological change can be inferred from seeding the coupled ocean–iceberg–sea-ice model with a constant, or unvarying, net annual iceberg flux from major tidewater glaciers and marine ice shelves around the Arctic. The coupled model then predicts well below the observed iceberg number crossing 48° N in most years (figure 2) and is poorly correlated with I48N (*r*=0.11). Variability in the marine environment through which icebergs travel therefore has little discernible impact on modelled I48N and is not the principal factor leading to the large observed variability in figure 2. This means that variations in the upper ocean dynamics caused by interannual variability in wind, and the air–sea heat and freshwater fluxes, and interannual variation in the melting rate of the iceberg, related to ocean temperature and wave strength, are insufficient to explain the highly variable I48N.

**Figure 4.**
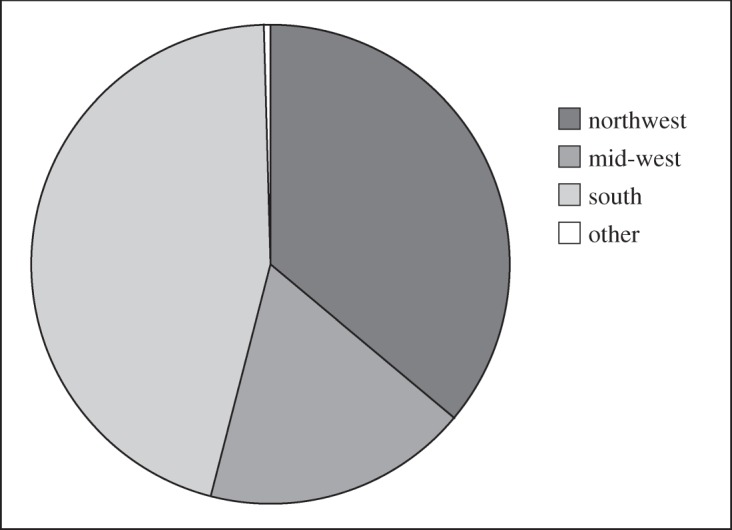
Source region for fine resolution model I48N, with variable yearly discharge, as determined by equation (2.1). Different regions are defined as follows. ‘South’ is from those sources equatorward of 65° N. ‘Mid-west’ denotes releases from between 65° N and 72° N on the west coast of Greenland or Baffin Island (although the latter do not in fact contribute to I48N). ‘Northwest’ refers to icebergs from all other release sites in Baffin Bay, Nares Strait and the Canadian Arctic islands north of 72° N. ‘Other’ is all of eastern Greenland above 65° N plus sites in Svalbard and the Russian Arctic islands.

If open ocean circulation and temperature change has not caused the dominant variability in iceberg numbers then there must be some fundamental relationship with iceberg discharge from sources whose icebergs reach 48° N. It is worth noting that this was the implication of a fjordal model study of iceberg sedimentation as well [46], where the glaciological regime was found to be more important than fjordal sea temperature. To confirm this, another experiment was performed, using the same atmospheric forcing, where the annual calving discharge for each Northern Hemisphere glacier was defined by equation (2.1). The resulting model prediction for I48N (variable discharge line in figure 2) shows very high correlation (*r*=0.83). From the ocean–iceberg modelling, it is found that over 99% of the modelled icebergs crossing 48° N originated from southern or western Greenland fjords, or other glaciers within northern Baffin Bay (figure 4). The ocean model results therefore strongly suggest that variation in Greenland glacial discharge should dominate the underlying fluctuation of I48N. As the largest changes in SMB in recent years have occurred in the ablation zone [47], and therefore around calving glaciers, it is likely that the relatively well-known SMB will be physically related to the poorly known discharge [3]. Previously, it has been shown that there is not a simple linear relationship between SMB and I48N [1]. A more complex, nonlinear relationship between this index, SMB, and parameterizations of regional climatic and local oceanic change, taken as the NAO and LSST, was therefore sought using NARMAX modelling.

### (c) Nonlinear auto-regressive moving average with exogenous modelling

**Figure 5.**
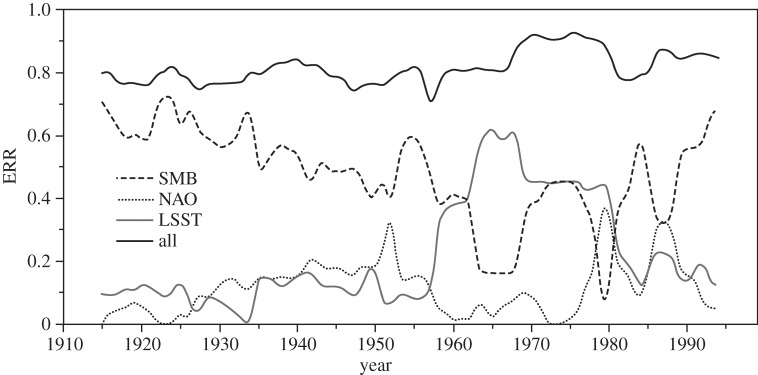
Contributions to the NARMAX model. Computed contributions of SMB, LSST and NAO to the annual iceberg numbers over 1900–2008, based on the ERR values for a 30 year sliding window, incremented 1 year at a time, where SMB, LSST and NAO were considered as inputs. The ERR value of each window for each variable was calculated by summing the ERR value of selected model terms that included the considered variable. While the model will never explain all the variance in the time series, owing to observational error and model simplifications, the sum of the contributions from SMB, LSST and NAO (always less than 1.0) is a measure of the model fit. To avoid inconsistencies due to initial and final conditions in the dataset, only those years centred in a distinct 30 year window are shown. The initial (before 1915) and final (after 1993) ERR contributions were ignored as the windows centred on these years would have been necessarily shorter than the standard 30 year window because of the finite data time series.

The results of the full NARMAX model of I48N are shown in figure 2 and demonstrate a high level of fit (*r*=0.84; see table 1 for characteristic ERR values, but it should be remembered that these are just two realizations of models from the 79 sliding window intervals). Figure 5 shows the evolving contributions of SMB, NAO and LSST to the variance explained by the NARMAX model. For the first half of the twentieth century, the contribution of SMB to the model, based on summing the ERR values, is strongly dominant, explaining 50–60% of the variance in I48N. There is then a period when the climatic indices of the NAO, and particularly LSST, explain similar, or greater, amounts of the variation in I48N compared with the SMB. However, in recent years, SMB has reverted to being dominant. This late twentieth century importance of the climate-related indices is not directly linked to climate change affecting iceberg trajectories, as the ocean model (figure 2) shows little change in the mean number of icebergs reaching 48° N over the last few decades, even though the NAO and LSST have generally been higher (figure 1).

**Table 1.** NARMAX model terms for two example sliding window periods of the 79 calculated.

sliding window 1900–1929		sliding window 1956–1985	
term	ERR	term	ERR
32.1SMB(*t*−4)	0.659	196SMB(*t*).LSST(*t*).LSST(*t*−1)	0.762
830LSST(*t*−4).LSST(*t*−3)^2^	0.095	3470NAO(*t*).NAO(*t*−3).LSST(*t*−2)	0.156
−0.0171SMB(*t*−2).SMB(*t*−3).SMB(*t*−4)	0.045	43.2SMB(*t*−1).NAO(*t*−2).LSST(*t*−4)	0.039

**Figure 6.**
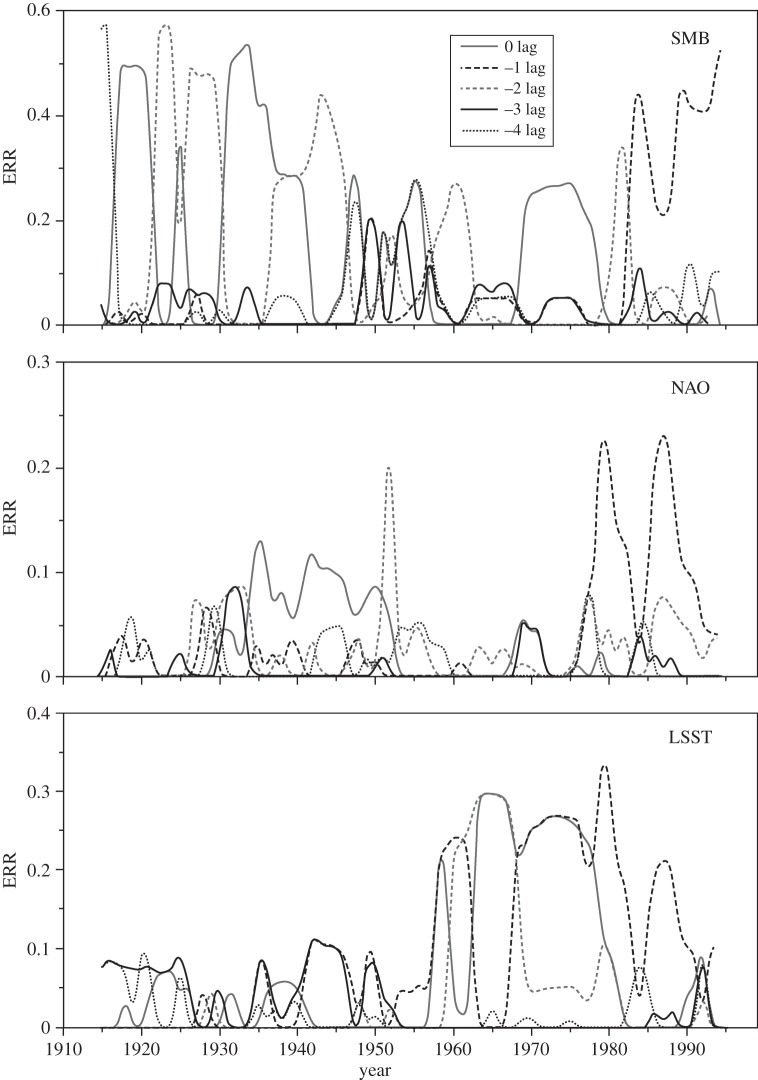
Computed contributions to the annual iceberg numbers over 1900–2008 of: (*a*) the lags of SMB, (*b*) NAO and (*c*) LSST. These lags are based on the ERR values for a 30 year sliding window, incremented 1 year at a time, where only one variable was considered as the input. To avoid inconsistencies due to initial and final conditions in the dataset, the initial (before 1915) and final (after 1993) ERR contributions were ignored because of insufficient samples.

To explore this further, the relative importance of the different time lags in the NARMAX model and how this varied during the twentieth century were examined (figure 6). For much of the twentieth century, SMB has an important component with no lag, alternating, until 1980, with one of 2 years. For the rest of the century, the 1 year lag becomes consistently important. By contrast, the LSST shows a mix of lags during the long period where its overall importance is small, although with more signal in longer lags, but then, when it becomes a major part of the model after the mid-1950s, a strong signal at 0, 1 and sometimes 2 year lags. The NAO's contribution has no lag when its contribution is strongest, during the 1930s to 1950s, but is generally mixed at other times, except for the last few decades, when a 1 year time lag is often dominant. Thus, in the first half of the twentieth century, annual variation in the late spring peak of I48N was controlled by the GrIS SMB of the previous winter, or that two winters before (see figure 6, SMB panel). As suggested by the example shown in table 1 for the sliding window 1900–1929, the dominant relationship at that time tended towards a simple, linear dependence on SMB. However, in the last few decades, the dominant influences have switched to a mix of nonlinear terms, with lags up to 2 years, but with major contributions from 0 and 1 year lags.

## 4. Discussion and conclusion

**Figure 7.**
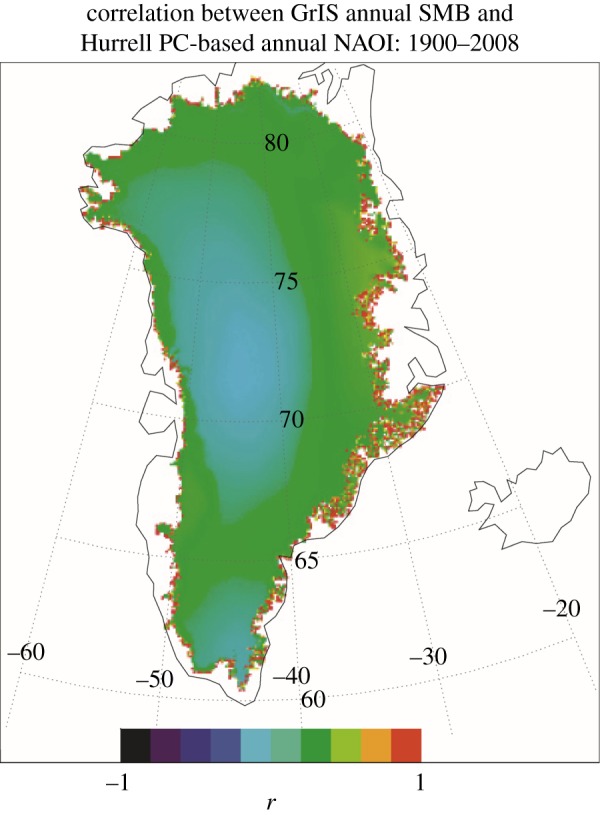
Correlation map of annual NAO with the local (5 km) annual SMB over 1900–2008. (Online version in colour.)

We hypothesize that the reason for the change in NARMAX model behaviour over the twentieth century, particularly the lag changes, is a combination of changing calving origin of those icebergs reaching 48° N, declining sea ice off Newfoundland [48] and warming sea surface temperatures in the Labrador Sea and Baffin Bay encouraging calving increases [15,49]. We propose that these factors may have led to a switch during the middle years of the century from an earlier, simpler, situation where icebergs tended to originate from southern Greenland (electronic supplementary material, figure S3), and hence reached 48° N quickly enough for there to appear to be no lag in annual terms, to one where there is a greater mix of icebergs originating from more distant western and northwestern Greenland (from where modelled travel times to 48° N are typically 1–3 years; see electronic supplementary material, figure S1), plus some still from southern Greenland. We suggest one reason for this change is the observed decline of about a third in maximum sea-ice area in the Labrador Sea [48] from 1920 to 1970, leading to more icebergs escaping from the sea ice in Baffin Bay, rather than a real change in calving origin. However, recent warming of the LSST (figure 1), and so increased sea temperatures near calving fronts [15,49], is likely to have led to a general increase in average calving rates. In addition, strong local correlation of GrIS SMB and NAO immediately up-glacier from the calving zones (figure 7) supports significant interaction between these variables at this local scale, through atmospheric variability, particularly affecting run-off, which has increased significantly in the last two decades [1], and perhaps basal sliding rates near the glacier front. The rise in importance, in models of I48N, of SMB in recent decades, shown in figures 5 and 6, is also consistent with a climatically driven increase in calving, through more run-off favouring faster outlet flow [50–52]. Combining these factors, it is likely that there has been a general increase in average calving rates in recent decades, as suggested by the I48N record of figure 2.

Combining the varied model and data sources used in this paper leads to the conclusion that I48N, even at an annual scale, is strongly related to iceberg calving from Greenland, and that the GrIS's SMB and the LSST are the major causal factors linked to this calving. The implication of this finding is that the GrIS calving discharge has increased quickly, but episodically, in recent decades, just as run-off has [53], and the SMB has been declining [1,2,47]. This is consistent with the episodic nature of retreat records from Jakobshavn Isbrae in west Greenland [7], sudden retreats at Kangerdlugssuaq and Helheim in east Greenland in 2004–2005 [8,14,15], and, more generally, the wide range of retreat rates for glaciers around Greenland during 2000–2010 [54]. From our analysis, it is difficult to separate out the variations over time of the regions which have contributed to this episodic flux. I48N cannot differentiate between sources. However, an indication is given by the coupled ocean–iceberg model variation of the origin of icebergs contributing to I48N. This is demonstrated in the electronic supplementary material, figure S2, which clearly shows the switch from southern dominance to a more mixed modelled source distribution from the 1930s onwards. In the last two decades, the model suggests a change from more important northwest Greenland sources to west Greenland sources. A comparison of marine core ice-rafted debris and modelled iceberg origins in the Denmark Strait, off east Greenland, has shown observational support for inferences from modelled iceberg origins [55]. However, it must be remembered that the imposed calving flux varies in the same way for a given year over the whole of Greenland in the model, so what we see in the electronic supplementary material, figure S3, is a convolution of glaciological and oceanic effects varying over time. Nevertheless, the sustained importance of 1 year lags for all variables in the NARMAX model in the last few decades (figure 6) supports the ocean–iceberg model result that this variation is most likely from west and south Greenland, rather than further north.

Our results suggest that I48N, suitably scaled, is an effective proxy for the GrIS calving flux, or at least for that from the southern and western quadrants of Greenland (figure 2). The series shows short periods of high flux throughout the last century, with particularly high fluxes during more extended periods centred around 1910, 1940, 1958, 1975 and 1985. The most distinctive part of the series, however, is the period since the early 1990s, with more than a decade of generally sustained high flux, but highly variable from year to year. This result can be compared with the distinctly smooth grounding line flux curves produced by van den Broeke *et al*. [47] and Rignot *et al*. [2]. These are likely to match calving discharge over longer timescales, but may be problematic in monitoring year-to-year calving variation, which will most likely be driven by local, calving conditions, as suggested by the wide range of retreat rates around Greenland in the last decade [54]. Decadal means of I48N, scaled as in the right-hand side discharge axis of figure 2, are given in table 2. This suggests that, in the mean, despite the large interannual variability, the GrIS in the first half of the twentieth century experienced similar levels of iceberg discharge leading to I48N. There was then a minimum during the 1950s and 1960s, with an increase from the 1970s onwards, peaking in the 1990s at levels similar to, but exceeding those reconstructed by Rignot *et al*. [2]. Notably, the most recent period (2000–2008) shows a decrease from these levels by over 50%, but still consistent with calving at an historically high level.

**Table 2.** Decadal means of reconstructed GrIS iceberg discharge over the twentieth century, from transforming I48N as shown in figure 2. Discharge is in km^3^ yr^−1^ or Gt. Standard deviation for each decade shown in brackets.

decade	discharge	decade	discharge
1900–1909	250 (204)	1960–1969	96 (85)
1910–1919	216 (185)	1970–1979	254 (340)
1920–1929	264 (209)	1980–1989	335 (407)
1930–1939	267 (172)	1990–1999	659 (345)
1940–1949	211 (228)	2000–2008	271 (238)
1950–1959	148 (187)		

The implicit assumption in table 2 is that east Greenland calving behaves in a similar fashion to that from west Greenland, and possible changes in the mean size of icebergs reaching 48° N reflect changes in tidewater glacier thickness as well as source region. However, even taking into account observational biases over time, this makes the recent past an unprecedented period for high GrIS iceberg fluxes, consistent with rapid changes in Greenland climate occurring around this time [1], but with significant interannual variability. This will result in a distinctly variable freshwater input to the northwest Atlantic Ocean, and so a more variable interannually changing effect on its circulation.

## References

[1] Hanna E 2011 Greenland ice sheet surface mass balance 1870 to 2010 based on twentieth century reanalysis, and links with global climate forcing. J. Geophys. Res. Atmos. 116, D24121 (doi:10.1029/2011JD016387)

[2] Rignot E Velicogna I Van den Broeke MR Monaghan A Lenaerts J 2011 Acceleration of the contribution of the Greenland and Antarctic ice sheets to sea level rise. Geophys. Res. Lett. 38, L05503 (doi:10.1029/2011GL046583)

[3] Rignot E Box JE Burgess E Hanna E 2008 Mass balance of the Greenland ice sheet from 1958–2007. Geophys. Res. Lett. 35, L20502 (doi:10.1029/2008GL035417)

[4] Bigg GR 1999 An estimate of the flux of iceberg calving from Greenland. Arct. Antarct. Alpine Res. 31, 174–178 (doi:10.2307/1552605)

[5] Krabill W 2004 Greenland ice sheet, increased coastal thinning. Geophys. Res. Lett. 31, L24402 (doi:10.1029/2004GL021533)

[6] Rignot E Kanagaratnam P 2006 Changes in the velocity structure of the Greenland ice sheet. Science 311, 986–990 (doi:10.1126/science.1121381) 16484490 10.1126/science.1121381

[7] Csatho B Schenk T Van der Veen CJ Krabill WB 2008 Intermittent thinning of Jakobshavn Isbrae, West Greenland, since the Little Ice Age. J. Glaciol. 54, 131–144 (doi:10.3189/002214308784409035)

[8] Andresen CS 2012 Rapid response of Helheim Glacier in Greenland to climate variability over the past century. Nat. Geosci. 5, 37–41 (doi:10.1038/ngeo1349)

[9] Murphy DL Cass JL 2012 The International Ice Patrol, safeguarding life and property at sea. Coast Guard Proc. Mar. Saf. Sec. Coun. 69, 13–16

[10] Christensen E Luzader J 2012 From sea to air to space, a century of iceberg tracking technology. Coast Guard Proc. Mar. Saf. Sec. Coun. 69, 17–22

[11] Howat IM Box JE Ahn Y Herrington A McFadden EM 2010 Seasonal variability in the dynamics of marine-terminating outlet glaciers in Greenland. J. Glaciol. 56, 601–613 (doi:10.3189/002214310793146232)

[12] Marko JR Fissel DB Wadhams P Kelly PM Brown RD 1994 Iceberg severity off eastern North America, its relationship to sea ice variability and climatic change. J. Clim. 7, 1335–1351 (doi:10.1175/1520-0442(1994)007<1335:ISOENA>2.0.CO;2)

[13] Wolford TC 1981 Sea ice and iceberg conditions, 1970–79. NAFO Sci. Coun. Stud. 5, 39–42

[14] Luckman A Murray T de Lange R Hanna E 2006 Rapid and synchronous ice-dynamic changes in East Greenland. Geophys. Res. Lett. 33, L03503 (doi:10.1029/2005GL025428)

[15] Seale A Chrisoffersen P Mugford RI O’Leary M 2011 Ocean forcing of the Greenland Ice Sheet, calving fronts and patterns of retreat identified by automatic satellite monitoring of eastern outlet glaciers. J. Geophys. Res. 116, F03013 (doi:10.1029/2010JF001847)

[16] Yashayaev I 2007 Hydrographic changes in the Labrador Sea, 1960–2005. Prog. Oceanogr. 73, 242–276 (doi:10.1016/j.pocean.2007.04.015)

[17] Berkson JM Allen AA Murphy DL Boda KJ 2010 Integrated Ocean Observing System (IOOS (R)) supports marine operations: a look from the US Coast Guard. Mar. Technol. Soc. J. 44, 156–165 (doi:10.4031/MTSJ.44.6.22)

[18] Wilton DJ Bigg GR Hanna E Submitted Modelling twentieth century global ocean circulation and iceberg flux at 48° N: implications for west Greenland iceberg discharge.

[19] Janssens I Huybrechts P 2000 The treatment of meltwater retention in mass-balance parameterisations of the Greenland ice sheet. Ann. Glaciol. 31, 133–140 (doi:10.3189/172756400781819941)

[20] Hurrell JW Deser C 2009 North Atlantic climate variability, the role of the North Atlantic oscillation. J. Mar. Syst. 78, 28–41 (doi:10.1016/j.jmarsys.2008.11.026)

[21] Kaplan A Cane M Kushnir Y Clement A Blumenthal M Rajagopalan B 1998 Analyses of global sea surface temperature 1856–1991. J. Geophys. Res. Oceans 103, 18567–18589 (doi:10.1029/97JC01736)

[22] Compo GP 2011 The Twentieth Century Reanalysis project. Q. J. R. Meteorol. Soc. 137, 1–28 (doi:10.1002/qj.776)

[23] Levine RC Bigg GR 2008 Sensitivity of the glacial ocean to Heinrich events from different iceberg sources, as modelled by a coupled atmosphere-iceberg-ocean model. Paleoceanography 23, PA4213 (doi:10.1029/2008PA001613)

[24] Wadley MR Bigg GR 2002 Impact of flow through the Canadian Archipelago on the North Atlantic and Arctic thermohaline circulation, an ocean modelling study. Q. J. R. Meteor. Soc. 128, 2187–2203 (doi:10.1256/qj.00.35)

[25] Bigg GR Dye SR Wadley MR 2005 Interannual variability in the 1990s in the northern Atlantic and Nordic Seas. J. Atmos. Ocean Sci. 10, 123–143 (doi:10.1080/17417530500282873)

[26] Bigg GR Wadley MR Stevens DP Johnson JA 1996 Prediction of iceberg trajectories for the North Atlantic and Arctic Oceans. Geophys. Res. Lett. 23, 3587–3590 (doi:10.1029/96GL03369)

[27] Bigg GR Wadley MR Stevens DP Johnson JA 1997 Modelling the dynamics and thermodynamics of icebergs. Cold Reg. Sci. Technol. 26, 113–135 (doi:10.1016/S0165-232X(97)00012-8)

[28] Baur A 1968 Nouvelle estimation du bilan de masse de l’Inlandsis du Groenland. Deep Sea Res. 14, 13–17

[29] Reeh N 1994 Calving from Greenland glaciers, observations, balance estimates of calving rates, calving laws. In Workshop on the calving rate of West Greenland glaciers in response to climate change (ed. Reeh N), pp. 85–102 Copenhagen, Denmark: Danish Polar Centre

[30] Gladstone R Bigg GR Nicholls KW 2001 Icebergs and fresh water fluxes in the Southern Ocean. J. Geophys. Res. 106, 19903–19915 (doi:10.1029/2000JC000347)

[31] Death R Siegert MJ Bigg GR Wadley MR 2006 Modelling iceberg trajectories, sedimentation rates and meltwater input to the ocean from the Eurasian Ice Sheet at the Last Glacial Maximum. Palaeogeogr. Palaeoclim. Palaeoecol. 236, 135–150 (doi:10.1016/j.palaeo.2005.11.040)

[32] Green CJ Bigg GR Green JAM 2010 Deep draft icebergs from the Barents Ice Sheet during MIS 6 are consistent with erosional evidence from Lomonosov Ridge, central Arctic. Geophys. Res. Lett. 37, L23606 (doi:10.1029/2010GL045299)

[33] Bigg GR Levine RC Green CL 2011 Modelling abrupt glacial North Atlantic freshening: rates of change and their implications for Heinrich events. Glob. Planet. Change 79, 176–192 (doi:10.1016/j.gloplacha.2010.11.001)

[34] Newell JP 1993 Exceptionally large icebergs and ice islands in eastern Canadian waters: a review of sightings from 1900 to present. Arctic 46, 205–211 (doi:10.14430/arctic1345)

[35] Valeur HH Hansen C Hansen KQ Rasmussen L Thingvad N 1996 Weather, sea and ice conditions in eastern Baffin Bay, offshore northwest Greenland: a review. Danish Meteorological Institute Technical Report 96–12, Copenhagen, Denmark.

[36] Chen S Billings SA 1989 Representations of non-linear systems: the NARMAX model. Int. J. Control 49, 1013–1032 (doi:10.1080/00207178908559683)

[37] Billings SA Chen S Korenberg MJ 1989 Identification of MIMO nonlinear systems using a forward regression orthogonal estimator. Int. J. Control 49, 2157–2189 (doi:10.1080/00207178908559767)

[38] Billings SA 2013 Non-linear system identification: NARMAX methods in the time, frequency, and spatio-temporal domains Chichester, UK: Wiley

[39] Nehmzow U Akanyeti O Billings SA 2010 Towards modelling complex robot training through system identification. Robot. Auton. Syst. 58, 265–275 (doi:10.1016/j.robot.2009.11.002)

[40] Balikhin MA Boynton RJ Walker SN Borovsky JE Billings SA Wei HL 2011 Using the NARMAX approach to model the evolution of energetic electrons fluxes at geostationary orbit. Geophys. Res. Lett. 38, L18105 (doi:10.1029/2011GL048980)

[41] Zhao Y Billings SA Wei HL Sarrigannis PG 2012 Tracking time varying causality and directionality of information flow using an error reduction test with applications to electroencephalography data. Phys. Rev. E 86, 051919 (doi:10.1103/PhysRevE.86.051919) 10.1103/PhysRevE.86.051919 23214826

[42] Straneo F 2013 Challenges to understanding the dynamic response of Greenland's marine terminating glaciers to oceanic and atmospheric forcing. Bull. Am. Meteorol. Soc. 94, 1131–1144 (doi:10.1175/BAMS-D-12-00100.1)

[43] Zhao Y Bigg GR Billings SA Hanna E Sole AJ Wei H Kadirkamanathan V Wilton DJ Submitted Inferring the variation of climatic and glaciological contributions to west Greenland iceberg discharge in the twentieth century.

[44] Kugiumtzis D 2000 Surrogate data test for nonlinearity including nonmonotonic transforms. Phys. Rev. E 62, R25–R28 (doi:10.1103/PhysRevE.62.R25) 10.1103/physreve.62.r25 11088516

[45] Schrieber T Schmitz A 2000 Surrogate time series. Physica D 142, 346–382 (doi:10.1016/S0167-2789(00)00043-9)

[46] Mugford RI Dowdeswell JA 2010 Modeling iceberg-rafted sedimentation in high latitude fjord environments. J. Geophys. Res. Earth Surf. 115, F03024 (doi:10.1029/2009JF001564)

[47] van den Broeke M Bamber J Ettema J Rignot E Schrama E van de Berg WJ van Meijgaard E Velicogna I Wouters B 2009 Partitioning recent Greenland mass loss. Science 326, 984–986 (doi:10.1126/science.1178176) 19965509 10.1126/science.1178176

[48] Hill BT Jones SJ 1990 The Newfoundland ice extent and the solar cycle from 1860 to 1988. J. Geophys. Res. 95, 5385–5394 (doi:10.1029/JC095iC04p05385)

[49] Holland DM Thomas RH De Young B Ribergaard MH Lyberth B 2008 Acceleration of Jakobshavn Isbrae triggered by warm subsurface ocean waters. Nat. Geosci. 1, 659–664 (doi:10.1038/ngeo316)

[50] Walsh KM Howat IM Ahn Y Enderlin EE 2012 Changes in the marine-terminating glaciers of central east Greenland, 2000–2010. Cryosphere 6, 211–220 (doi:10.5194/tc-6-211-2012)

[51] Zwally HJ Abdalati W Herring T Larson K Saba J Steffen K 2002 Surface melt-induced acceleration of Greenland ice-sheet flow. Science 297, 218–222 (doi:10.1126/science.1072708) 12052902 10.1126/science.1072708

[52] Hewitt IJ 2013 Seasonal changes in ice sheet motion due to melt water lubrication. Earth Plant. Sci. Lett. 371, 16–25 (doi:10.1016/j.epsl.2013.04.022)

[53] Hanna E Huybrechts P Steffen K Cappelen J Huff R Shuman C Irvine-Fynn T Wise S Griffiths M 2008 Increased run-off from melt from the Greenland Ice Sheet, response to global warming. J. Clim. 21, 331–341 (doi:10.1175/2007JCLI1964.1)

[54] Howat IM Eddy A 2011 Multi-decadal retreat of Greenland's marine-terminating glaciers. J. Glaciol. 57, 389–396 (doi:10.3189/002214311796905631)

[55] Andrews JT Bigg GR Wilton DJ In press Holocene ice-rafting and sediment transport from the glaciated margin of east Greenland (67–70° N) to the N Iceland shelves: detecting and modelling changing sediment sources. Q. Sci. Rev. (doi:10.1016/j.quascirev.2013.08.019)

